# Determinants of Length of Stay After Vaginal Deliveries in the Friuli Venezia Giulia Region (North-Eastern Italy), 2005–2015

**DOI:** 10.1038/s41598-020-62774-6

**Published:** 2020-04-06

**Authors:** L. Cegolon, G. Maso, W. C. Heymann, M. Bortolotto, A. Cegolon, G. Mastrangelo

**Affiliations:** 1 Local Health Unit N.2 “Marca Trevigiana”, Public Health Department, Veneto Region, Treviso, Italy; 2 Institute for Maternal & Child Health, IRCCS “Burlo Garofolo”, Trieste, Italy; 3 Florida State University, Department of Clinical Sciences, College of Medicine, Sarasota, Florida USA; 4 Florida Department of Health, Sarasota County Health Department, Sarasota, Florida USA; 5 Padua University, FISPPA Department, Padua, Italy; 6 University of Macerata, Department of Political, Social & International Relationships, Macerata, Italy; 7 Padua University, Department of Cardio-Thoracic & Vascular Sciences, Padua, Italy

**Keywords:** Epidemiology, Paediatric research

## Abstract

Although length of stay (LoS) after childbirth has been diminishing in several high-income countries in recent decades, the evidence on the impact of early discharge (ED) on healthy mothers and term newborns after vaginal deliveries (VD) is still inconclusive and little is known on the characteristics of those discharged early. We conducted a population-based study in Friuli Venezia Giulia (FVG) during 2005–2015, to investigate the mean LoS and the percentage of LoS longer than our proposed ED benchmarks following VD: 2 days after spontaneous vaginal deliveries (SVD) and 3 days post instrumental vaginal deliveries (IVD). We employed a multivariable logistic as well as a linear regression model, adjusting for a considerable number of factors pertaining to health-care setting and timeframe, maternal health factors, newborn clinical factors, obstetric history factors, socio-demographic background and present obstetric conditions. Results were expressed as odds ratios (OR) and regression coefficients (RC) with 95% confidence interval (95%CI). The adjusted mean LoS was calculated by level of pregnancy risk (high vs. low). Due to a very high number of multiple tests performed we employed the procedure proposed by Benjamini-Hochberg (BH) as a further selection criterion to calculate the BH p-value for the respective estimates. During 2005–2015, the average LoS in FVG was 2.9 and 3.3 days after SVD and IVD respectively, and the pooled regional proportion of LoS > ED was 64.4% for SVD and 32.0% for IVD. The variation of LoS across calendar years was marginal for both vaginal delivery modes (VDM). The adjusted mean LoS was higher in IVD than SVD, and although a decline of LoS > ED and mean LoS over time was observed for both VDM, there was little variation of the adjusted mean LoS by nationality of the woman and by level of pregnancy risk (high vs. low). By contrast, the adjusted figures for hospitals with shortest (centres A and G) and longest (centre B) mean LoS  were 2.3 and 3.4 days respectively, among “low risk” pregnancies. The corresponding figures for “high risk” pregnancies were 2.5 days for centre A/G and 3.6 days for centre B. Therefore, the shift from “low” to “high” risk pregnancies in all three latter centres (A, B and G) increased the mean adjusted LoS just by 0.2 days. By contrast, the discrepancy between maternity centres with highest and lowest adjusted mean LoS post SVD (hospital B vs. A/G) was 1.1 days both among “low risk” (1.1 = 3.4–2.3 days) and “high risk” (1.1 = 3.6–2.5) pregnanices. Similar patterns were obseved also for IVD. Our adjusted regression models confirmed that maternity centres were the main explanatory factor for LoS after childbirth in both VDM. Therefore, health and clinical factors were less influential than practice patterns in determining LoS after VD. Hospitalization and discharge policies following childbirth in FVG should follow standardized guidelines, to be enforced at hospital level. Any prolonged LoS post VD (LoS > ED) should be reviewed and audited if need be. Primary care services within the catchment areas of the maternity centres of FVG should be improved to implement the follow up of puerperae undergoing ED after VD.

## Introduction

At the beginning of the 20th century home births were the norm and hospital deliveries very rare. Women started to deliver in hospital during World War 2 (WW2), in facilities near the military areas where their respective partners were training. This trend continued in the decades following WW2, with standard length of stay after childbirth (LoS) increasing up to 10 days^1,2^.

In the 70 ies some USA hospitals started to assess the health of mothers and newborn for eligibility to return home within 12–24 hours after childbirth, with a midwife on call for domiciliary care up to 3 days for 2 weeks post discharge^1^.

In 1992 the American Academy of Pediatrics (AAP) and the American College of Obstetricians and Gynecologists (ACOG) formalized the most frequently shared definition of early discharge (ED) after childbirth worldwide as a LoS less than 48 h post spontaneous vaginal deliveries (SVD) and less than 96 h post cesarean section (CS)^3^. Thereafter the reduction of LoS expanded to other high-income countries, with increasing applications of ED^4^.

LoS after childbirth remained however a controversial aspect of obstetric care, creating an open debate not only on its impact on the health of mothers and babies but also on health policies, state legislations and functioning of the respective health care systems. Nevertheless, ED of mothers and newborn has in fact increased dramatically in several high-income countries over the past 10–15 years. However, the evidence on the impact of ED on healthy mothers and term newborns (≥37 weeks) after a vaginal delivery (VD) is still inconclusive and little is known of the characteristics of those discharged early^5–13^.

Since LoS has become a critical indicator of efficiency of health care delivery, understanding its associated factors could provide information helpful in the reduction of health care costs, improvement in the delivery of obstetric care, containment of untoward events associated with comorbidities and complications requiring readmission^14,15^. For instance, in Canada (excluding Quebec) from 2003 to 2010, neonatal readmission rates were lowest for LoS of 1–2 days following VD and 2–4 days after CS^16^.

Several factors are reportedly associated with LoS in the open literature, including readiness for discharge (clinical and perceived) of the mother^8,17–19^. However, information on the impact of medical/obstetrical conditions associated with pregnancies is scarce or totally lacking.

Using a comprehensive database with information on a considerable number of factors, we previously reviewed the case mix of hospital performance by LoS post SVD as well as instrumental vaginal deliveries (IVD) during 2005–2015 in Friuli Venezia Giulia (FVG), a region of North-Eastern Italy^6^.

In this study we present the impact of the outstanding determinants on LoS following SVD and IVD, with the aim of informing health care policy makers.

## Methods

The methods have been reported in previous papers^6,7^ and are herewith briefly described.

### Study design

This is a population-based cross-sectional study to investigate LoS after VD during 2005–2015 in FVG. The study was approved by the Regional Health Authority of FVG, a regional governmental body issuing anonymized health data routinely collected by the Italian National Health Service (NHS) to research institutions within the frame of approved protocols/studies, overseeing also their use is in compliance with the current Italian privacy regulations (D.Lgs 101/2018).

### The database

Data from the 12 maternity services of FVG during calendar years 2005–2015 were extracted from the Regional Repository, a database anonymously storing administrative information from the Italian National Health Service (NHS). The database we analyzed included information from two sources: hospital discharge forms (ICD-9 codes) and the Certificate of Delivery Care (CEDAP, Italian acronym), a formatted questionnaire recording extensive (clinical and personal) data on new mothers and their babies^6,7,20,21^.

We used the following ICD-9 codes to retrieve the obstetric conditions associated with each childbirth:

- Polyhydramnios: 657.0;
- Oligohydramnios: 658.0;
- Antepartum hemorrhage, abruptio placentae and placenta previa: 641.(0-1-2-3-8-9);
- Obstructed labour (but shoulder girdle dystocia): 660.(0-1-2-3-5-6-7-8-9);
- Non-reassuring fetal status: 656.3;
- Cord prolapse: 663.0;
- Premature rupture of membranes (PROM): 658.1;
- Eclampsia/pre-eclampsia: 624.(4-5-6-7);
- Rh iso-immunization: 656.1;

The rest of data derived from CEDAP, in which the delivery mode is defined as follows^6,7,20,21^:

1. Vaginal delivery (VD) without forceps or vacuum extraction;
2. Planned CS (PCS) or CS for failed induction;
3. CS during labour or urgent CS;
4. Forceps extraction;
5. Vacuum extraction;
6. Other forms of VD.

For the purpose of this study, we used the above categories 1 as SVD, whereas categories 4,5 and 6 were assembled into IVD.

The 12 regional facility centres were anonymized and coded by alphabetic letter from A to L. However, maternity units of FVG were 11 (A to K). L is a major regional university hospital where only a limited number of complicated deliveries were referred during the study period. Consequently, this centre was excluded from analysis. A and B are second level maternity units (>1,000 annual births and equipped with an neonatal intensive care unit), whereas the other 9 are first level (<1,000 annual births and/or devoid an neonatal intensive care unit)^6,7,20,21^.

**Figure 1:**
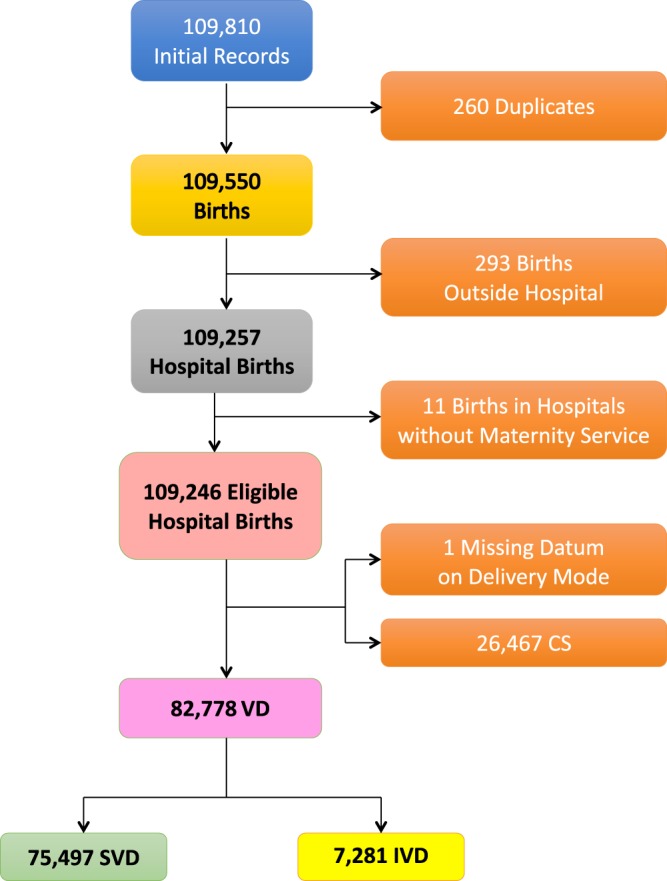
Flowchart displaying the various criteria applied to the initial database to obtain the final number of hospital records available for the analysis. CS = caesarean sections; IVD = Instrumental vaginal deliveries; SVD = spontaneous vaginal deliveries.

Figure 1 shows the flowchart displaying the various selection criteria applied to the initial eligible births (N = 109,246) to obtain the final number of SVD (=75,497) and IVD (=7,281) during 2005–2015 for the analysis.

### Length of Hospital Stay after childbirth

LoS (measured in days) was calculated by subtracting the date of birth from the date of hospital discharge, as previously described^6^. We considered the average LoS and the percentage of LoS > ED benchmarks^6,7^:

- 2 days following spontaneous vaginal deliveries (SVD);
- 3 days following instrumental vaginal deliveries (IVD).

**Figure 2:**
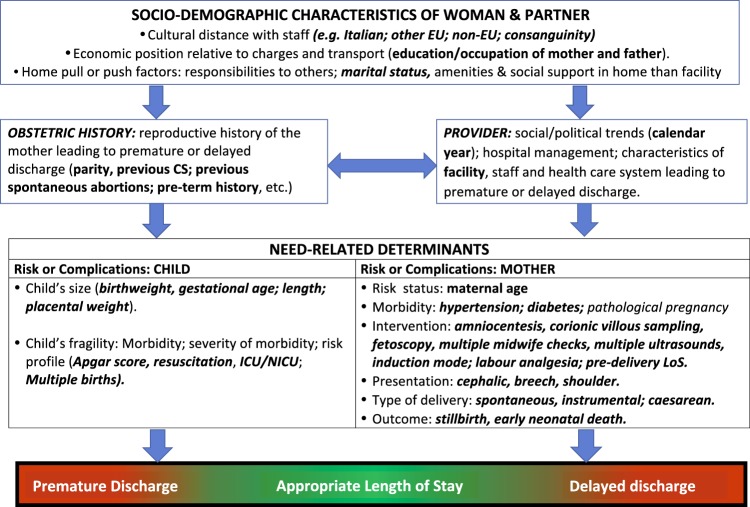
Conceptual Framework, displaying the relationship between various explanatory factors and length of hospital stay (LoS) after childbirth.

We employed the conceptual framework already adopted, identifying five broad domains of potential determinants of LoS (Fig. 2)^6,7^.

**Figure 3:**
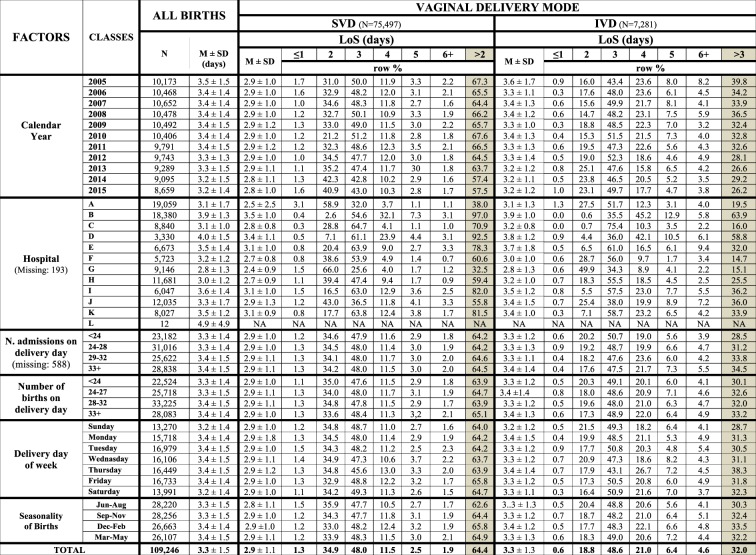
Distribution of Length of Stay (LoS) after childbirth by maternity centre and calendar year. Number (N), row percentage (%); mean LoS (M) ± standard deviation (SD); M = missing values; NA = Not Applicable. SVD = Spontaneous vaginal delivery; IVD = instrumental vaginal delivery.

**Figure 4:**
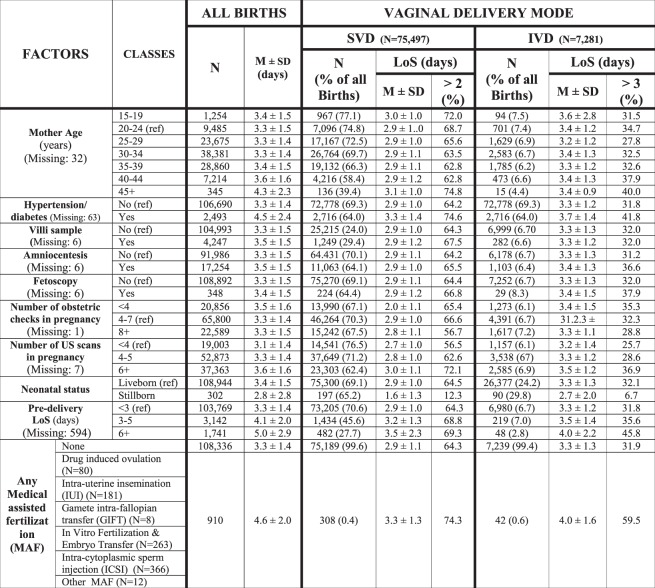
Distribution of Length of Stay (LoS, in days) after childbirth by maternal health factors. Number (N), row percentage (%); mean LoS (M) ± standard deviation (SD); M = missing values.

**Figure 5:**
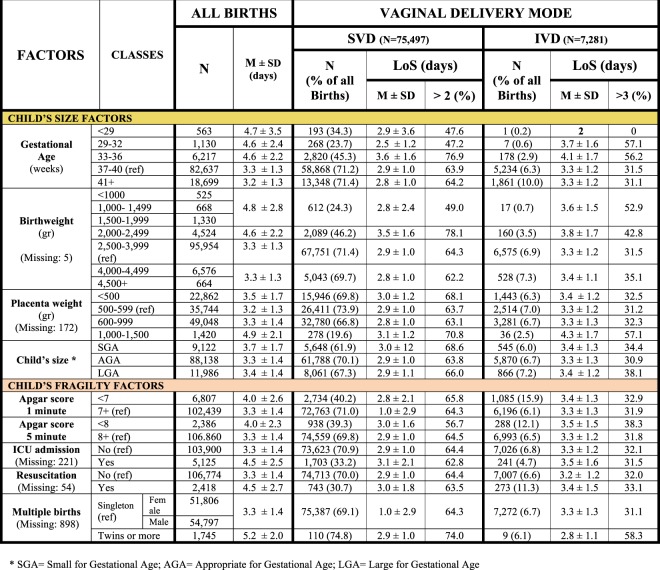
Distribution of Length of Stay (LoS, in days) after childbirth by clinical factors of the child. Number (N), row percentage (%); mean LoS (M) ± standard deviation (SD); M = missing values.

**Figure 6:**
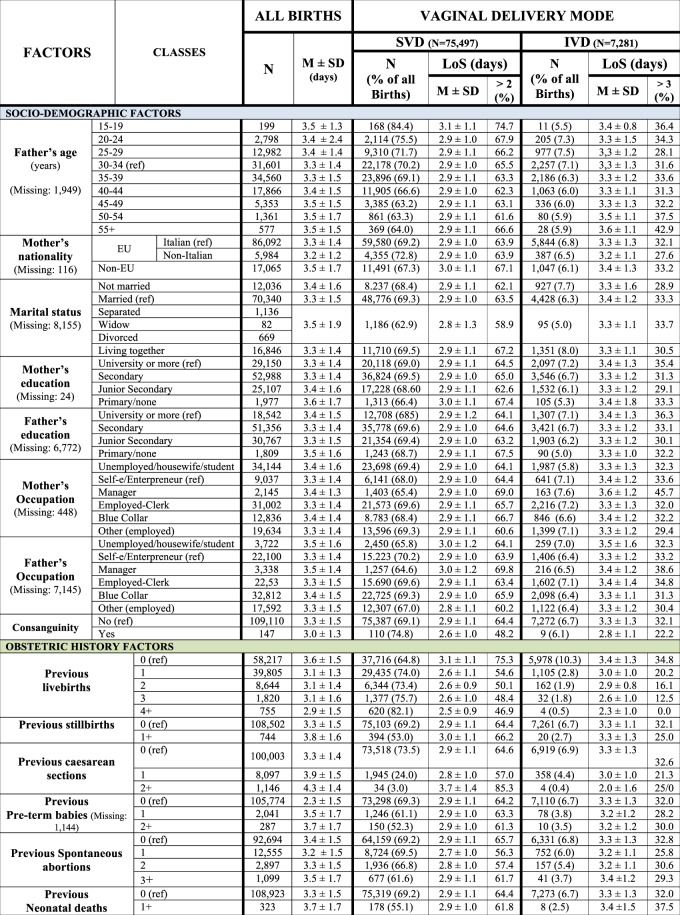
Distribution of Length of Stay (LoS, in days) after childbirth by socio-demographic and obstetric history factors. Number (N), row percentage (%); mean LoS (M) ± standard deviation (SD).

**Figure 7:**
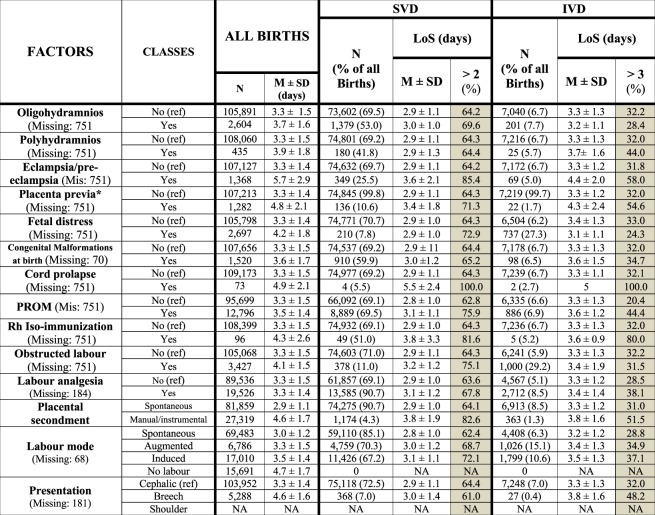
Distribution of Length of Stay (LoS, in days) after childbirth by obstetric factors. Number and row percentage (%). Mean LoS (M) ± standard deviation (SD). SVD = Spontaneous vaginal deliveries; IVD = instrumental vaginal deliveries. NA = not applicable.

1. Health care setting and timeframe: hospitals and calendar year (Fig. 3).
2. Maternal health factors (Fig. 4): mother’s age; hypertension/diabetes; amniocentesis, villous sampling; fetoscop; pre-delivery LoS; presentation; infant survival status; number of obstetric checks performed in pregnancy, number of ultrasound (US) scans performed during pregnancy; any medical assisted fertilization.
3. Clinical factors of the child (Fig. 4), in particular:
  1. Child’s size factors: gestational age; birthweight; placenta weight; and a variable “child’s size” created combining the distribution of four factors: sex of child; number of previous libebirths; birthweight and gestational age. The variable “child’s size” enabled us to classify a newborn into small for gestational age (SGA); appropriate for gestational age (AGA); and large for gestational age (LGA)^6,7,21–24^.
  2. Child’s fragility factors: Apgar score at 1 minute; Apgar score at 5 minutes; resuscitation; intensive care unit (ICU) admission; multiple births.

1. Socio-demographic background (Fig. 5), namely: mother’s nationality; marital status of the mother; mother’s education; mother’s occupation; father’s age; father’s education; father’s occupation; consanguinity.
2. Obstetric history (Fig. 6): number of previous livebirths; number of previous CS; number of previous stillbirths; number of previous pre-term births; number of previous spontaneous abortions; number of previous neonatal deaths.
3. Obstetric conditions (Fig. 7): oligohydramnios; polyhydramnios; eclampsia/pre-eclampsia; placenta previa/abruptio placenta/ante-partum hemorrhage; non reassuring fetal status;  congenital malformations at birth; cord prolapse; premature rupture of membranes (PROM); Rh Iso-immunization; obstructed labour (but girdle dystocia); labour analgesia; labour mode; fetal presentation.

### Statistical analysis

As a rule of thumb, when there were minor mismatches between the two sources of information we had for this study (CEDAP and HDF), priority was given to CEDAP. Therefore, the original data have been modified as follows:

- In CEDAP fetal presentation is classified as follows: vertex; breech; shoulder; face; brow; bregma; other. The group “other” was excluded from the analysis and coded as missing. Vertex, face, brow and bregma were grouped into a comprehensive class named “cephalic”. As already described, 39 shoulder presentations delivered by SVD and 1 shoulder presentation delivered by IVD were reclassified as cephalic^6^.
  The mean LoS and the percentage of LoS longer than the proposed ED benchmarks following SVD (2 days) and IVD (3 days) were calculated for each of the above explanatory factors. We chose the most widely adopted ED benchmarks for SVD. We proposed a 3 days ED threshold for IVD, as a compromise between SVD (2 days) and CS (4 days) cutoffs recommended by the AAP and the ACOG^3^. The 0/1 variable LoS (lower/higher than ED) was used as a dichotomous outcome in two multiple logistic regression models.
  Some factors were deliberately removed from the final multivariate logistic and linear regression model for the following reasons:
- Resuscitation, due to collinearity with intensive care unit (ICU), and (only for IVD) Apgar score at 1 minute, due to collinearity with Apgar score at 5 minutes;
- father’s education, father’s occupation, marital status and pre-term history, since being affected by many missing values.

We fitted a multiple logistic regression model for each DM (SVD as well as IVD), using LoS as a binary endpoint (LoS > ED vs. LoS ≤ ED). Results were expressed as odds ratio (OR) with 95% confidence interval (95%CI). Additionally, we built up one multiple regression model for each DM (SVD as well as IVD), using LoS as a linear outcome.

Stepwise backward selection of independent variables was used to build up all four final regression models (linear as well as logistic), using p < 0.05 as a criterion. Results were expressed as regression coefficients (RC) or odds ratio (OR), with 95% confidence interval (95%CI). Results of all multiple regression models were obtained by comparing each stratum specific estimate (OR and RC) with the corresponding reference category.

In absence of technical indicators featuring non-interventional deliveries, we defined “low risk” pregnancies as those simoultaneously meeting as all the following conditions: no hypertension/diabetes of the mother; no assisted medical fertilization; singleton pregnancies; no eclampsia/pre-eclampsia; cephalic presentation; livebirth; pre-delivery LoS < 3 days; no oligohydramnios; no polyhydramnios; Apgar score at 5 minute ≥ 8; gestation 37–40 weeks; birthweight 2,5–4.0 Kg; placenta weighing 500–599 g; spontaneous labour; no administration of labour analgesia; no fetal distress; no obstructed labour; no PROM; spontaneous placental secondment; no cord-prolapse; no Rh iso-immunization.

We also defined “high risk” pregnancies, characterized by at least either of the following conditions: any assisted medical fertilization; multiple births; eclampsia/pre-eclampsia; breech/shoulder presentation; stillbirth; pre-delivery LoS ≥ 3 days; oligohydramnios; polyhydramnios; Apgar score at 5 minute <8; gestation <37 or ≥41 weeks; birthweight <2,5 or ≥4.0 Kg; placenta weighing <500 g or ≥600 g; labour induced or augmented; administration of labour analgesia; non-reassuring fetal status; obstructed labour; PROM; manual/instrumental placental secondment; cord-prolapse; Rh iso-immunization.

From the final multivariable linear regression models we then calculated the individual adjusted mean LoS by VDM, level of pregnancy risk (high vs. low), mother nationality (EU vs. non-EU) and maternity centre. Likewise, we also calculated the adjusted probability of LoS > ED by VDM and maternity centre.

Considering the large number of statistical tests performed in the multivariable logistic as well as linear regression models, some p-values could have been significant by chance. Therefore, we employed as a further selection criterion the procedure proposed by Benjamini-Hochberg (BH), setting the false discovery rate at 5% to obtain the BH p-value to be associated with each estimate (OR and RC)^24^.

Missing values were excluded, and complete case analysis was performed.

Stata 14.2 (College Station, Texas, USA) was employed for the analysis.

## Results

Figure 3 displays the distribution of LoS (mean and proportion of LoS > ED) after SVD as well as IVD by calendar year and hospital. The pooled mean LoS in FVG during 2005–2015 was 3.3 days, consistently exceeding our proposed ED benchmark for SVD in all maternity centres. Likewise, LoS exceeded ED for IVD in all maternity centres but G. Although a decreasing pattern of LoS > ED was observed for both VDM from 2005 to 2015 (more pronounced for IVD), the respective mean LoS remained steady over the years.

There was considerable variability of mean LoS and LoS > ED across all 12 hospitals for SVD, ranging from 32.5% in centre G up to 97.0% in centre B. Similar figures applied also to IVD. A high percentage of LoS > ED was generally accompanied by greater mean LoS in the various maternity centres. For instance, for SVD the highest unadjusted mean LoS were found in centres B (3.5 ± 1.0 days), D (3.4 ± 1.1 days), I (3.1 ± 1.0 days) and K (3.1 ± 0.9 days), which corresponded also to highest proportion of LoS > ED (97.0% for B, 92.5% for D, 82.0% for I and 81.5% for K). Likewise, for IVD higher crude mean LoS were found in hospital B (3.9 ± 1.0 days), D (3.8 ± 1.2 days), I (3.5 ± 1.2 days) and J (3.4 ± 1.5 days), whose respective proportions of LoS > ED were 63.9%, 58.8%, 36.2% and 36.0%. By contrast, hospital G had the lowest crude mean LoS post SVD (2.4 ± 0.9 days) as well as IVD (2.8 ± 1.3 days), with the respective proportion of LoS > ED being 32.5% for SVD and 15.1% for IVD. The proportion of LoS > ED was higher on Friday (65.8%) for SVD and on Thursday (38.3%) for IVD and for both VDM was slightly higher in the winter months (December-February).

As can be seen from Fig. 4, the variability of mean LoS and proportion of LoS > ED among categories was limited for each independent variable (especially hypertension/diabetes, mother’s age, presentation, pre-delivery LoS, and infant survival status).

As shown in Fig. 5, a higher average LoS and a greater proportion of LoS > ED corresponded to gestations of 33–36 weeks, birthweight 2,000–2,500 g and placental weight <500 g and ≥ 1,000 g. Among child’s fragility factors, multiple birth showed higher mean LoS and proportion of LoS > ED.

Figures of mean LoS and proportions of LoS > ED were rather balanced across socio-demographic factors of parents, while major differences were observed for obstetric history factors. Higher mean LoS was found in nulliparas for both VDM, whereas LoS post SVD was much longer in women with history of ≥2 CS (Fig. 6).

Figure 7 shows rather stable figures in mean LoS and crude rates of surpassing the ED cutoffs by obstetric conditions. As can be seen, among SVD higher mean LoS was found for cord prolapse, Rh iso-immunization, seconded placenta; eclampsia/pre-eclampsia; obstructed labour and labour induction and PORM. Among IVD, longer mean LoS corresponded to eclampsia/pre-eclampsia, placental secondment, breech presentation, polyhydramnios, PROM and labour induction.

The results of multivariable logistic regression (OR, 95%CI and BH p-value) for factors associated with LoS longer than our proposed ED benchmarks for both SVD and IVD are shown in Supplementary Table 1a. As can be seen, for SVD a significant decrease of OR for LoS > ED was found with increasing calendar year. Moreover LoS > ED was less considerably likely with ≥1 previous livebirths, whereas it was significantly higher for induced labour, non-EU nationality of the mother, manual/instrumental placental secondment, gestational age 33–36 weeks, maternal age ≥35, CS history, eclampsia/pre-eclampsia, birthweight 2,000–2,500 g and ≥4,000 g, hypertension/diabetes and multiple births. By contrast, stillbirth, gestation <32 weeks and very low birthweight (<2,000 g), were all associated with a significant reduction of LoS > ED, probably because the latter three factors were correlated with each other. Among delivery days of the week, the adjusted probability of LoS > ED for SVD was higher on Fridays.

The ORs were largely overlapping between SVD and IVD, with the corresponding estimates being similar though milder for IVD. Among IVD LoS > ED was more likely with pre-term gestations (33–36 weeks), eclampsia/pre-eclampsia, manual/instrumental secondment of placentas, labour analgesia and Apgar score at 5 minutes <8. Furthermore, Thursdays, labour induction, placental weight 600–999 and Rh iso-immunization all tended to significantly increase the probability of LoS > ED.

Supplementary Table 1b diplays the adjusted risk estimates hor hospitals, controlled for the same factors of the two multivariable logistic models, whose results are indicated at the bottom of Supplementary Table 1a. As can be seen, all maternity services were rather consistently more likely to surpass the the ED cutoffs for SVD as compare with the reference (centre A). The level of significance of hospital estimates was remarkably higher than any other factors examined in Supplementary Table 1a, clearly suggesting that hospital variability was by far the main determinant of the probability of LoS > ED.

Supplementary Table 2a shows the results of the multiple linear regression models (RC, 95%CI and BH p-value) for SVD and IVD. The number of complete case observations was almost the same between the two regression models (logistic and linear). Whenever OR was lower than unity (Supplementary Table 1a), RC was a negative estimate (Supplementary Table 2a), with little difference between the two models (logistic vs. linear). RC, expressed in days, was generally less than 1, except for stillbirth on SVD (RC = −1.67), eclampsia/pre-eclampsia on SVD (RC = 1.03) and 4+ previous livebirths on IVD (RC = −1.56). Therefore, the presence/absence of health conditions of the mother and the newborn did not translate into marked variation of LoS.

As can be seen from Supplementary Table 2b, adjusted hospital estimates were by far the strongest determinants of longer mean LoS at multivariable linear regression, with all hospitals but G showing greater adjusted average LoS post SVD as compared with the reference (centre A). The pattern of mean LoS post IVD was similar, although the respective adjusted estimates were somewhat less significant and the adjusted mean LoS of two centres (B and F) ended up not being significantly different from A (the referen thospital).

**Figure 8:**
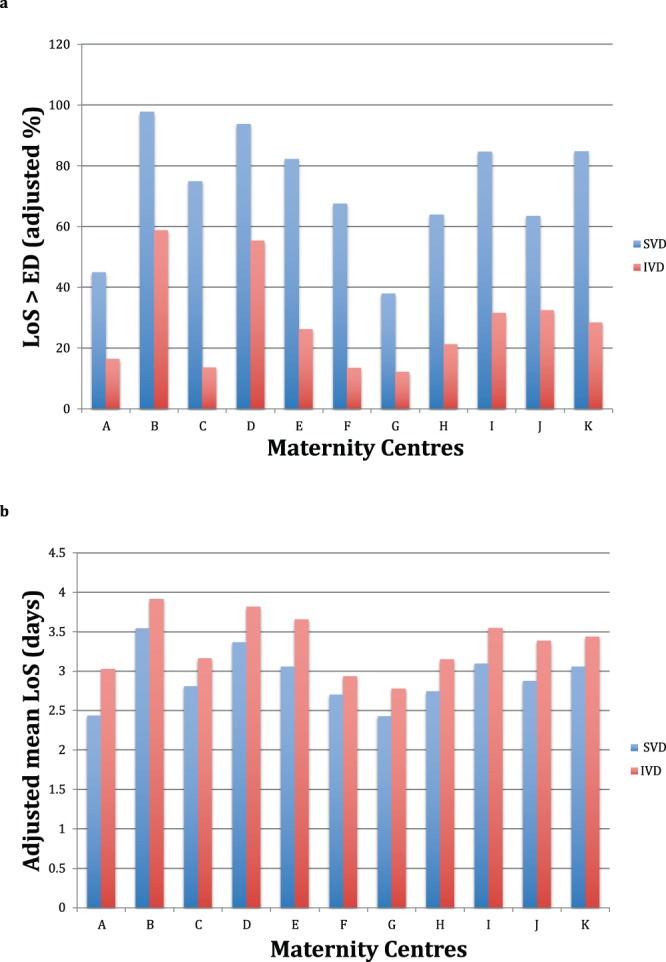
(**a**) (upper pannel of Figure 8). Adjusted proportions of length of hospital stay (LoS) > early discharge (ED) for spontaneous vaginal deliveries (SVD; ED = 2 days) and instrumental vaginal deliveries (IVD; ED = 3 days) in Friuli Venezia Giulia (FVG) during 2005–2015, by maternity centre. Estimates adjusted for the same factors displayed at the bottom of Supplementary Table 1a,b. (**b**) (lower pannel of Figure 8). Adjusted mean of length of hospital stay (LoS; in days) after spontaneous vaginal deliveries (SVD) and instrumental vaginal deliveries (IVD) in Friuli Venezia Giulia (FVG) during 2005–2015, by maternity centre. Estimates adjusted for the same factors displayed at the bottom of Supplementary Table 2a,b.

Figure 8a shows a bar chart with the adjusted rates of LoS > ED in FVG during the entire study period, by VDM and hospital, controlled for all explanatory factors of the respective multiple loigistic regression models, listed at bottom of Supplementary Table 1a,b (See Supplementary File). Likewise, Fig. 8b displays the adjusted mean LoS in FVG during the study timeframe, by VDM and maternity centre, controlled for the same factors of the corresponding multiple linear regression models, displayed at the bottom of Supplementary Table 2a,b (see Supplementary File).

**Figure 9:**
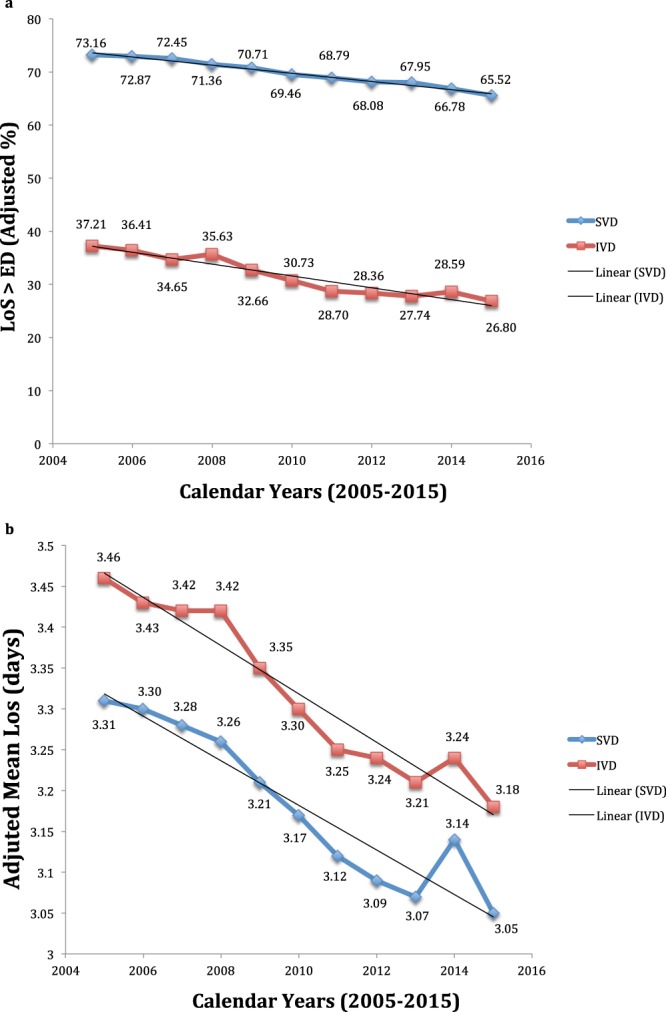
(**a**) (upper pannel of Figure 9). Adjusted rates of length of hospital stay (LoS) > early discharge (ED) benchmarks after Spontaneous Vaginal Deliveries (SVD, ED cutoff = 2 days) and Instrumental Vaginal Deliveries (ED cutoff = 3 days) in Friulia Venezia Giulia over time (2005–2015). Estimates adjusted for the same factors displayed at the bottom of Supplementary Table 1a,b. (**b**) (upper pannel of Figure 9). Adjusted mean length of hospital stay (LoS) after spontaneous vaginal delivery (SVD) and instrumental vaginal delivery (IVD) in Friulia Venezia Giulia (FVG) over time (2005–2015). Estimates adjusted for the same factors displayed at the bottom of Supplementary Table 2a,b.

Figure 9a,b show the rates of LoS > ED and the mean LoS following both VDM in FVG over time, adjusted for the same factors of the corresponding multiple linear regression models, displayed at the bottom of Supplementary Table 2a,b (see Supplementary File). As can be seen a regionwide progressive delcine in both estimates over the years.

As can be noted from Supplementary Table 3, the adjusted mean LoS post SVD (controlled for the same factors displayed at the bottom of Supplementary Table 2a,b) was 2.7 days in “low risk” pregnancies among EU women and 2.8 days in non-EU women. Considering “high risk” pregnancies, the mean LoS after SVD was 3.1 days among EU women and 3.3 days among non-EU women. Therefore, the presence of risky health conditions for the mother and/or the newborn increased LoS after SVD by 0.3 days both in EU women (difference = 3.0–2.7 days) and non-EU women (difference = 3.1–2.8 days). The corresponding figures for hospitals with shortest and longest adjusted mean LoS were 2.3 days (A and G) vs. 3.4 days (centre B), respectively for “low risk pregnancies”. Among “high risk” pregnancies the latter estimates increase to 2.5 days in centres A and G and to 3.6 days for hospital B (Supplementary Table 3). Therefore the shift from “low” to “high” risk pregnancies enhanced LoS post SVD just by 0.2 days both in centres A and G (difference = 2.5–2.3 days) and B (difference = 3.6–3.4 days). By contrast, the discrepancy between maternity centres with highest and lowest adjusted mean LoS post SVD (hospital B vs. A/G) was 1.1 days both among “low risk” (difference = 3.4 – 2.3 days) and “high risk” (difference = 3.6–2.5 days) pregnancies.

As can be seen from Supplementary Table 3, consistent marginal enhancements of 0.1–0.2 days shifting from “low” to “high” risk pregnancies were also observed for LoS after IVD, although in case of EU women in hospitals B, D J and K this increase was slightly higher (0.3 days). The differences between maternity centres with lowest (hospital G) and highest (hospital B) adjusted mean LoS post IVD was 0.9 days (=3.6–2.7) for low risk and 1.3 days (=3.9–2.7) for high risk gestations, respectively.

Figure 9a diplays the adjusted proportions of LoS > ED for SVD as well as IVD in FVG over time, adjusted for the same factors displayed at the bottom of Supplementary Table 1a,b.

Figure 9b diplays the adjusted mean LoS for SVD and well as IVD in FVG over time, adjusted for the same factors displayed at the bottom of Supplementary Table 2a,b. As can be noted, there was a decrease of the proportion of LoS > ED and adjusted mean LoS for both VDM.

## Discussion

### Key findings

In the whole FVG region during 2005–2015, the average LoS was 2.9 days for the 75,497 SVD and 3.3 days for the 7,281 IVD (Fig. 3). The pooled probability of surpassing the ED benchmarks (2 days post SVD and 3 days post IVD) was double for SVD (64.4%) as compared to IVD (32.0%) in the entire region during the study period (Fig. 3). This evidence suggests that a benchmark of 3 days for LoS post IVD may be an over-estimation. Following hospital, the most influential determinants of ED post SVD were higher number of previous livebirths, labour induction, stillbirth, increasing calendar year, mother nationality, manual/instrumental placental secondment, gestational age 33–36 weeks, maternal age 35–44 years and eclampsia-pre-eclampsia. For IVD the main factors were 1–2 previous livebirths, pre-term gestations, eclampsia/pre-eclampsia, manual/instrumental placental secondment and labour analgesia (Supplemengary Tables 1a and 2a).

Gestational age <32 weeks was highly correlated with both stillbirth (p < 0.001) and birthweight (p < 0.001), and presumably for this reason the probability of LoS > ED was significantly lower for all these three factors.

### Strengths and limitations

This is a population-based study where the high number of records available for the analysis enabled substantial statistical power and accuracy of results. The dataset analyzed included a considerable number of factors that may affect LoS and is trustworthy, since data were recorded by trained health care staff^6,7^. The highly adjusted estimates of the present study enable to draw evidence based conclusions to format policies, practices and actions to evaluate obstetric care at regional as well as national level. As far as we are aware there are no published studies investigating LoS after VD using such a structured, comprehensive and detailed methodology.

There are however some limitations.

The primary limitation of our study concerns the estimation of ED based upon days rather than hours of hospitalization. It would certainly be more appropriate to use the exact time of birth and hospital discharge for the calculation of LoS. Unfortunately, this was not possible with CEDAP, as the questionnaire records information on time of birth but date of hospital discharge. Further, the variable “time of birth” was affected by a considerable number of missing values in our database (42.6% = 46,577/109,246). Nevertheless, the use of day metrics instead of hours for the calculation of LoS was little relevant in our study, since we found LoS for physiological and uncomplicated births being 2.7–2.8 days rather than 12 or 24 hours. Furthermore, although it would be certainly interesting to know the exact LoS in hours (especially for its impact on the wellbeing of the woman and her family and for the actual daily occupancy rate of hospital beds), in Italy only the number of hospitalization days are relevant for the calculation of the respective health care costs. There is also wide variability in the discharge time across FVG maternity units, and it would likely be difficult to standardized and convert this information into actual days of hospital stay.

A second limitation of our study is the proportion of missing values affecting some relevant factors such as father’s education, father’s occupation and marital status, which had to be dropped from the final regression models. The distribution of missing values for all these factors was not random, hence we felt imputation to be inappropriate. In addition to reluctance of the woman to disclose some personal information, it may also be the case that socio-demographic information was felt to be less important than clinical information by the staff filling up CEDAP questionnaire.

We did not have information on the council of residence of the new mother in this study, which would have allowed to assess the impact on LoS of distance of woman’s residence from the designated maternity unit.

Lastly, for years 2005–2015 CEDAP did not record important maternal information reportedly associated with LoS: smoking; body mass index (BMI); physical activity; bleeding during delivery (in ml); confidence of the mother with breastfeeding and her readiness for discharge.

### Interpretation of findings

A physiological birth generally requires few or no interventions^25^. Whilst risky conditions of the mother and/or the newborn increased LoS post SVD by 0.2 days for both EU and non-EU mothers, the difference between maternity centres with highest and lowest adjusted mean LoS after SVD was 1.1 days both for “low” as well as “high” risk pregnancies. Likewise, the corresponding hospital differences in LoS post IVD were 0.9 days for “low risk” and 1.3 days for “high risk” pregancies. These features were also supported by the results of the multiple linear regression analysis, where the vast majority of RC were smaller than 0.5.

Althoug a marginal decrease of LoS > ED and mean LoS for both VDM was observed in whole region, maternity centres were the strongest determinant for LoS after adjustment for maternal health factors, newborn’s clinical factors, obstetrical history, socio-demographic background and major medical/obstetric conditions. Medical factors were therefore less influential than practice patterns in determining LoS after VD. This was also somehow confirmed also by the lack of association of LoS with both regionwide number of births and admissions on delivery day, an indication that decision making on time of discharge was probably not influenced by the bed turn-over and the contigency capacity of the various maternity units. The remarkable variability of LoS > ED and mean LoS across regional maternity services of FVG for both VDM was already reported^6^. Similar conclusions on Los variability by hospital were also reported from Canada^26^ and Denmark^18^.

The marginal decreasing LoS observed for both VDM over time at regional level suggests growing perception among the medical community and society that extended hospitaliztions following uncomplicated births are unnecessary. However, this trend was limited, since a structured and systematic system of primary care services following up the dyad woman-newborn after ED is still lacking in FVG (and in Italy in general).

In the present study LoS was also longer during the winter (December-February) as well as spring (March-May) months for SVD, an indication that possibly cold weather and higher risk of cold related co-morbidities may have pushed clinicians to retain women slightly more in hospital.

Interestingly, LoS was also more likely to overtake the ED cutoff for SVD on Fridays, whereas among IVD LoS > ED was more likely on Thursdays. In Italy the civil registration offices are shut over the week-end, therefore women potentially eligible to be discharged on Saturday or Sunday are instead retained in hospital until Monday, when their child can be registered at the local city council.

### Generalizability

#### Early discharge

The term “early discharge” describes a hospital discharge occurring before the usual or customary time^17^. In the present study the ED benchmark was 2 days for SVD and 3 days for IVD. The United States (US) legislation enacted mandatory insurance coverage for a minimum postpartum LoS of 48 hours for mothers and babies after VD^27^. However, the US legislation left the ultimate decision on timing of discharge on the new mother and her caregiver^17^. In a recent survey conducted in Denmark on 2,786 women, about 60% (=1,620) were discharged less than 50 hours after birth, one third (=934) very early (<12 hours) and 85% of the latter group within 6 hours^18^. In another recent study carried out in the Province of Trento (North-Eastern Italy) on 52,957 newborns delivered during 2006–2016, employing an ED cutoff of 3 days for SVD and 5 days for CS, an increase of ED following VD from 10.4% in 2006 up to 52.4% in 2016 was observed^19^. Similar loose ED benchmarks (3 days for SVD and 5 days for CS) were also applied in a French study of 128,382 births recruited from the Sentinel AUDIPOG network during 1994–2002, reporting an increasing ED after VD from 3% in 1997 up to 7% in 2002. Altough the latter French study estimated that 40% primaparas and 55% multiparas could be potentially discharged early in France^28^. The variable definition of ED and the health status of the mother and newborn complicate its appraisal in terms of health impact.

Furthermore, although generally it is the best method to evaluate the effect of an intervention, a randomized controlled trial (RCT) design is problematic and impractical for ED, because of poor recruitment and crossover of study subjects, hence researchers are generally discouraged from pursuing RCTs on LoS^29^. Much of the evidence on the impact of ED therefore comes from observational studies using routine hospital data or information from healthcare insurance claims, with no adequate control for confounders^29^. As a result, the evidence  on safety of ED on mother and newborn health is still inconclusive.

Nevertheless, an ED policy could intuitively have several positive advantages: on woman’s health/comfort, breast-feeding, her family as well as on the efficiency of health systems.

#### Impact of ED on health systems

One of the principal aims of ED is the provision of cost-effective postnatal care by decreasing unnecessary days of LoS^30,31^. In a RCT on 459 Swiss pregnant women without complications, the cost of ED followed by domiciliary care was significantly lower than standard discharge (SD)^32,33^. Similar evidence was found in another RCT on 430 Spanish pregnant women tested for ED within 24 h post-partum followed-up by home care; in the latter study a reduction of 20% costs in the ED program was observed.

In addition to higher efficiency of health services, both latter RCTs reported no significant differences between ED and SD in terms of neonatal and maternal morbidity and psychological health, with a 90% satisfaction rate among women undergoing ED, and an even higher rate of breast feeding at 3 months in the ED arm^34^. The absence of psychological implications from ED was confirmed also by another European RCT^33^. Likewise, a Canadian RCT reported that 97% women experiencing ED expressed satisfaction with the home care service received and were happy to return earlier to the comfort and privacy of their home^35^. In the same Canadian study, 60% interviewed women rated longer LoS more appropriate for primiparas feeling unprepared and concerned about an early return home^35^. The above findings contrast with Brown’s, who reported that women discharged within 48 h post-partum were at higher risk of depression 5–6 months after childbirth^4^.

It is argued that ED might delay a prompt detection and treatment of maternal and infant complications^36,37^. Readmission rates have therefore been considered to investigate the impact of LoS. The most recent Cochrane systematic review on 10 RCTs comparing postnatal ED with SD showed no significant differences between ED and SD in terms of infant readmissions to hospital or other important outcomes^4^. Several large retrospective cohort studies from high-income countries reported maternal readmissions within 28 days following birth for postnatal bleeding, retained products of conception, infection and postnatal mental health^29,38^. In addition, neonatal readmission rates for dehydration, gastro-enteritis, jaundice, and scarce weight gain have been investigated^36,39–45^. Some of these readmissions might be the result of an insufficient time to assess and support the puerpera immediately after child delivery^4,33,46^.

Other studies reported a number of maternal disorders following ED, namely fatigue, insomnia, depression, fear, stress, breast-feeding issues, and constipation^43,47,48^. In 42% cases these disturbances required access to health services^47^.

Supporters of ED claim it is safe, it reduces the risk of hospital acquired infections, promotes family bonding/attachment and contain health care cost associated with hospitalization. However, ED also has some potential downsides, which have been stressed^36^:

1. The establishment of breastfeeding is delayed until the third postpartum day or even later;
2. Some conditions do not present until 2 or more days following delivery;
3. Time is reduced for in-hospital counselling on breastfeeding, infant care and women’s health.

According to Braveman “*the currently available literature provides little scientific evidence to guide discharge planning for most apparently well newborns and their mothers*”^38^. Other authors reported higher risk of morbidity and readmissions of infants within their first 14 days of life being associated with shorter LoS^49^ . A 22% higher risk of readmission in the first 28 days of life was quantified in relation to discharges of healthy infants within 30 hours following birth^50^

#### Determinants of ED

ED is reportedly associated with a number of factors, including multi-parity^17,28,51,52^, advanced maternal age^52^, nationality/ethnicity^17,51^, labour analgesia, labour induction, cigarette smoking, body mass index (BMI), marital status, parental occupational status, multiple birth, infant survival status, DM, type of birth attendant^8,18,19^, normal birthweight (2500–5000 g), term gestational age (38–40 weeks)^52^, formula feeding of the infant at discharge, others^17,52^. Additional factors associated with ED are reportedly lower socio-economic status (SES)^51^, public payment^17^ and a lower community deprivation score^53^. Variability of LoS post VD by hospital has been already reported and may be due to a number of reasons^54^.

Although number of previous livebirths was the main determinant of ED in our study, the mean LoS of multiparas still exceeded the ED cutoffs for both SVD as well as IVD. The association between multi-parity and ED is consistent with the open literature^17,18,37,55,56^, and in particular with an Australian study reporting a growing proportion of primaparas undergoing ED increased from calendar year 1989 (rate of 1%) to 2000 (10%)^4^.

Leaving aside stillbirth, ED was associated with both labour induction and labour analgesia in our study. In particular, we found that the impact of labour analgesia on ED was positive for SVD but negative for IVD. Labour stimulating medications, epidural analgesia and perinatal bleeding greater than 500 ml decrease the chance of ED and are of critical importance, since the above three factors may be either the cause or the consequence of complicated deliveries^18,57^. Further, both labour induction and analgesia reportedly interferes with readiness for breastfeeding in the newborn, prolonging LoS^58^. The recourse to epidural painkillers has been increasing over the past two decades, with 24% women delivering in Denmark during 2013 administered with epidural analgesia^59^.

Higher maternal age (for SVD), hypertension/diabetes (for SVD), eclampsia/pre-eclampsia (for both VDM) were all associated with lower probility of ED in this study. A number of conditions as obesity, hypertension, diabetes, spontaenous abortion, pre-term delivery, bleeding and stillbirth are more likely with maternal age > 35^60–62^. Hypertension was associated with extended LoS for both VD and CS in a study of 1,015,424 livebirths from California during 2008–2009^15^. Further factors associated with extended LoS post VD in the latter US study were puerperal infections and transfusions. Other obstetrical factors influencing LoS post SVD in the present investigation were multiple births, PROM, oligohydramnios, non-reassuring fetal status and Rh iso-immunization (also associated with increased LoS post IVD). Maternal comorbidities and obstetric conditions require a higher level of care most often resulting in longer LoS^15^. Earlier recognition of these conditions facilitates their management, improves health outcomes of mother and newborn and may also reduce LoS^15^.

Another factor importantly associated with LoS > ED for both VDM in our study was late pre-term gestation (33–36 weeks). The risk of morbidity is significantly higher among immature neonates^63^, who also present higher need of hospital care and higher risk of readmission following ED, especially if breastfed^64^. An inception of breast-feeding before discharge from hospital is critical to prevent postanatal morbidy and readmissions.

#### Socio-demographic factors and perceived readiness of discharge

Studies considering a vast range of potential determinants, including also social, educational and phychological factors influencing breast-feeding are scarce or missing.

In a study in the Mid-Western part of the US on 1,192 mothers discharged within 2 days following uncomplicated VD, LoS was longer among older women, those with higher SES and educational level, primiparas, breastfeeding, white ethnicity, married or living with the child’s father, with family support and with a private payor source^17^. By contrast, ED was more likely with younger age, parity, public payor source, low SES, lack of readiness of the mother for discharge, bottle-feeding and absence of a neonatal clinical problem^17^.

In a recent study conducted in four out of five regions of Denmark during 2013–2014 on 2,786 women, higher odds of ED in the regions of North Denmark and Zealand were confirmed for younger mothers; however, differently from the above US study, primiparas were more likely to be discharged early^18^. Interestingly, the latter two Danish regions, characterized by the least restrictive ED policies, showed the highest odds for very early discharge (North Denmark) and ED (Zealand)^18^

As mentioned above, ED was found more likely with public payer source and low SES in the US^17^. Whilst the association between low SES on ED has been reported also elsewhere^37,52^, in the US it might be explained by unequal access to health services^17,37,52^. In Denmark, a country with universal access to health care, 70% mothers expected a LoS < 48 hours and almost 60% of the study population was discharged within 50 hours^18^. In Denmark obstetric care comprehensively considers the health, social and psychological status of the woman^58,65^. For instance, mothers of younger age and lower SES are discharged later, as they are less likely to start and continue breastfeeding^66^.

In the Zealand region (Denmark) women undergoing ED were more likely to be single, uneducated, smokers and with higher BMI^18^. Consistent with the latter Danish study, we found ED being less likely with lower maternal education^18^. This finding was confirmed in a study examining postnatal care among heathy newborns in 19 US States during 2000^56^. One of the plausible interpretations of this finding is that younger and/or less educated women may be more reluctant to start breastfeeding, hence they are more likely to remain longer in hospital after childbirth^66^. However, another recent Italian study conducted in the Trentino region (North Eastern Italy) did not find any association between educational level of the woman and LoS^19^.

Another study described smoking in relation to ED but could not find more smokers in the group staying 0–1 nights as compared to those admitted for more than 3 nights^67^. The impact of smoking on ED may be explained by smokers’ need to go outside hospital to smoke. These women might be keener to be discharged early in order to be free to smoke wherever and whenever they wish. Therefore, the needs and wishes of new mothers should be considered in the decision process regarding postnatal LoS^18^. The woman’s expectation on LoS, her social/family support and previous breastfeeding experiences/knowledge were factors significantly associated with ED after childbirth in the above Danish study^18^. Likewise, in the above investigation on 1,192 women from the Mid-Western part of the US socio-demographic factors and readiness for discharge (objective and perceived) were also significantly associated with LoS^17^.

Since LoS is influenced by multiple aspects related to the woman, her family, the provider and the health system, decision making on timing of discharge should also include the perceived readiness for discharge of the new puerpera^17^. The identification of women with special needs requiring extended hospitalizations should be discussed between obstetricians and their patients^18^. Satisfaction of the woman towards the obstetric care received and her perceived sense of confidence post discharge may be influenced by her involvement in the decision making on timing of discharge^18^. This was confirmed in a recent meta-analysis, reporting the importance and interplay of perceived postnatal safety and sense of responsibility as key factors in building the confidence in own parental role^18^. In addition to parental satisfaction, involuntary ED and unreadiness for discharge are factors significantly associated also with fatigue, breastfeeding issues, poor health status requiring subsequent access to health care services^18,29,68–73^.

The implementation of structured readiness assessments during discharge preparation can assist clinicians in framing risk-mitigating actions for patient needs and reducing the likelihood of their hospital readmission. The use of a clinical assessment instrument to evaluate patient readiness for discharge has been recommended as an additional tool to standard care for discharge preparation^69,70^.

Similar to Nilsson^18^ and McMahon^74^, socio-demographic factors overall were not important factors in our analysis, with the exception of non-EU nationality of the mother (associated with lower probability of ED for SVD) and father’s age 20–29 years (less likely to be associated with ED for both VD modes). Similar figures were observed in the above mentioned study conducted in the Province of Trento (North-eastern Italy), where Italian women were more likely to be discharged earlier than non-Italian, although by only a small margin^19^. Younger fathers are more likely to be partners of younger mothers, who in turn are less likely to undergo pathological pregnancy courses and/or therefore to be retained in hospital longer as precautionary measure.

The significant role of mother’s nationality is confirmed in the open literature, although with conflicting figures. For instance, in a California study of 2,828 low-risk singleton births, ED was more likely for low income women, with untimely follow up more likely among Hispanics, non-English speaking women^55^. In another survey on 19 US states lower odds of ED were reported among black and Hispanic mothers^56^. This finding is interesting, since one may reasonably argue that in countries such as the US, ethnical minorities may have unequal access to health care for a number of reasons^17,37,52^, whereas in countries with universal health coverage such as Italy, less family support at home for foreign born as compared to native women may explain longer LoS.

Additionally, cultural factors may also be implicated in LoS, especially considering that in our study a high proportion of non-EU women were from eastern European countries (especially Albania, Kosovo, Serbia, Moldova and Bosnia Herzegovina), where the mean LoS post SVD is reportedly the longest within the European continent, probably being influenced by previous communist health policies still persisting nowadays^6^.

## Conclusions

Maternity centres remained a major driver of LoS for both VDM in FVG during 2005–2015, after controlling for the effect of maternal health factors, infant clinical factors, obstetric history, socio-demographic background and major medical/obstetric conditions. Therefore, health and clinical factors were less influential than practice pattern in determining LoS after VD.

Hospitalization and discharge policies following childbirth in FVG should follow standardized guidelines, to be enforced at hospital level^6,7^. Any prolonged LoS post VD (LoS > ED) should be reviewed and audited if need be. Primary care services within the catchment areas of the maternity centres should be improved to implement the follow up of puerperae undergoing ED after VD^6,7^. This process of change would require cultural preparation for both health care providers, users and the whole society.

## Supplementary information

Supplementary Information.

## Data Availability

This study analyzed third party data extracted from the Regional Repository of Friuli Venezia Giulia (FVG), a database anonymously storing potentially sensitive information. Access to this database is therefore subject to permission from the Regional Health Authority of FVG. Contact: Epidemiology & Health Information Service; Central Health Directorate; Health & Social Integration; Social & Family Policies; Via Pozzuolo 330, 33100, Udine, Italy. Tel: +39 0432 805661; email: salute@certregione.fvg.it.
