# Supplementary material for: Determinants of Length of Stay After Vaginal Deliveries in the Friuli Venezia Giulia Region (North-Eastern Italy), 2005–2015
Source: Sci Rep. 2020 Apr 6;10:5912. doi: 10.1038/s41598-020-62774-6 (PMC7136236; doi:10.1038/s41598-020-62774-6)
Supplement: Supplementary file 1 — Supplementary Information. [file 41598_2020_62774_MOESM1_ESM.pdf]

Determinants of Length of Stay After Vaginal Deliveries in the Friuli Venezia Giulia Region (North-Eastern Italy), 2005-2015

Cegolon L,[1,2] Maso G,[1] Heymann WC,[3,4] Bortolotto M,[5] Cegolon A,[6] Mastrangelo G[7]

1. Local Health Unit N.2 "*Marca Trevigiana*", Public Health Department, Veneto Region, Treviso, Italy

2. Institute for Maternal & Child Health, IRCCS "*Burlo Garofolo*", Trieste, Italy

3. Florida State University, Department of Clinical Sciences, College of Medicine, Sarasota, Florida, USA

4. Florida Department of Health, Sarasota County Health Department, Sarasota, Florida, USA

5. Padua University, FISPPA Department, Padua, Italy

6. University of Macerata, Department of Political, Social & International Relationships, Macerata, Italy

7. Padua University, Department of Cardio-Thoracic & Vascular Sciences, Padua, Italy

Correspondence
Dr. Luca Cegolon, MD, MSc, PhD
Local Health Unit N.2 "*Marca Trevigiana*"
Public Health Department
Treviso, Italy
Email: l.cegolon@gmail.com

Supplementary Table 1a. Multivariable logistic regression model for the probability of length of stay (LoS) > ED for spontaneous vaginal deliveries (SVD, 2 days cutoff) and instrumental vaginal deliveries (IVD, 3 days cutoff). Odds ratio (OR) with 95% confidence interval (95%CI); Benjamini Hochberg (BH) p-value set at 5% discovery rate (bottom of each cell). Obs.= complete (case analysis) observations.

**FACTORS (Reference category)**	**CLASSES**	**OR (95%CI) BH p-value**	
		**SVD** (72,786 obs.) **LoS > 2 vs. LoS ≤ 2**	**IVD** (7,020 obs.) **LoS > 3 vs. LoS ≤ 3**
Calendar year (2005-2015)	Linear term	0.95 (0.95; 0.96) 4.74E-47	0.97 (0.95; 0.99) 0.0033
Delivery day of week (Reference= Monday)	Thursday	NS	1.34 (1.08; 1.65) 0.0216
	Friday	1.11 (1.04; 1.19) 0.0046	NS
Seasonality (Reference: June-August)	September-Novermber	1.08 (1.03; 1.14) 0.0034	NS
	December-February	1.19 (1.13; 1.26) 3.45E-11	NS
	March-May	1.17 (1.12; 1.24) 1.72E-09	NS
Number of previous livebirths (Reference=0)	1	0.25 (0.24; 0.26) 0	0.39 (0.33; 0.47) 8.43E-22
	2	0.16 (0.15; 0.17) 0	0.24 (0.14; 0.39) 5.78E-08
	3	0.14 (0.12; 0.16) 1.60E-163	0.19 (0.05; 0.67) 0.0261
	4	0.16 (0.13; 0.20) 2.64E-66	NS
Number of previous CS (Reference =0)	1	1.47 (1.31; 1.64) 5.22E-11	NS
Mother nationality (Reference = Italian)	Non-EU	1.36 (1.28; 1.45) 1.36E-22	NS
Mother's age (Reference = 20-24 years)	25-29	1.10 (1.01; 1.18) 0.0347	0.74 (0.59; 0.94) 0.0325
	30-34	1.22 (1.12; 1.33) 6.25E-06	NS
	35-39	1.46 (1.33; 1.60) 1.64E-15	NS
	40-44	1.71 (1.52; 1.93) 4.80E-18	NS
	45+	3.90 (2.38; 6.40) 1.83E-07	NS
Mother educational level (Reference=University/more)	Primary/none	1.21 (1.04; 1.42) 0.0224	NS
Mother's occupation (Reference=unemployed/ student/housewife)	Self-employed/enterpreneur	0.90 (0.84; 0.97) 0.0133	NS
	Employed (other)	0.94 (0.88; 0.99) 0.0393	NS
Father's age (Reference =30-34 years)	20-24	0.83 (0.73; 0.95) 0.0096	NS
	25-29	0.90 (0.84; 0.96) 0.0038	0.74 (0.60; 0.90) 0.0095
Gestational age (Reference = 37-40 weeks)	<29	0.21 (0.12; 0.36) 9.87E-08	NS
	33-36	1.76 (1.57; 1.98) 5.47E-21	2.54 (1.77; 3.65) 3.06E-06
	41+	0.93 (0.89; 0.98) 0.0069	NS
Birthweight (Reference = 2.5-4.0 Kg)	<2.0	0.38 (0.27; 0.53) 4.97E-08	NS
	2.0-2.5	1.57 (1.37; 1.79) 2.93E-10	NS
	>4.0	1.17 (1.09; 1.26) 7.09E-05	NS
Multiple birth (Reference= singleton)	Twins or more	2.35 (1.60; 3.46) 3.53E-05	NS

Placenta weight (Reference = 500-999g)	<500	1.07 (1.01; 1.12) 0.0224	NS
	600-999	1.07 (1.02; 1.11) 0.0045	1.20 (1.05; 1.36) 0.0217
	1,000-1,500	1.64 (1.19; 2.26) 0.0041	NS
Hypertension/diabetes (Reference = No)	Yes	1.46 (1.24; 1.73) 2.28E-05	NS
Eclampsia/pre-eclampsia (Reference = No)	Yes	2.97 (2.11; 4.20) 1.63E-09	3.44 (1.97; 5.99) 6.50E-05
N. obstetric checks in pregnancy (Reference = 4-7)	<4	0.92 (0.87; 0.97) 0.0037	NS
N. US scans in pregnancy (Reference < 4)	6+	1.07 (1.01; 1.13) 0.0385	NS
Chorionic villous sampling (Reference = No)	Yes	1.13 (1.02; 1.25) 0.0277	NS
Rh iso-immunization (Reference = No)	Yes	3.11 (1.39; 7.00) 0.0100	21.93 (2.23; 216.10) 0.0237
Labour mode (Reference = Spontaneous)	Induced	1.57 (1.48; 1.66) 4.79E-54	1.21 (1.05; 1.39) 0.0262
	Augmented	1.15 (1.06; 1.24) 0.0009	NS
Labour analgesia (Reference = No)	Yes	0.86 (0.82; 0.91) 4.57E-08	1.22 (1.07; 1.38) 0.0090
Neonatal status (Reference = Liveborn)	Stillborn	0.02 (0.01; 0.03) 8.50E-45	NS
Premature rupture of membranes (Ref = No)	Yes	1.08 (1.02; 1.15) 0.0235	NS
Non reassuring fetal status (Reference = No)	Yes	1.75 (1.22; 2.49) 0.0045	NS
Obstructed labour (Reference = No)	Yes	1.63 (1.23; 2.14) 0.0012	NS
Apgar score at 5 minutes (Reference = 8+)	< 8	NS	1.52 (1.14; 2.03) 0.0155
Placental secondment (Reference=spontaneous)	Manual/ instrumental	2.53 (2.09; 3.06) 4.22E-21	1.80 (1.39; 2.32) 4.29E-05
Congenital Malformations at Birth: Present	Absent	1.21 (1.02; 1.43) 0.0400	
Placenta previa/abruptio p./ante-partum haemorrhage: Yes	No	1.65 (1.04; 2.61) 0.0463	

Results of Supplementary Tables 1a and 1b belong to the same model of multivariable logistic regression models, adjusted for the following factors:

SVD:

- **Health care setting and timeframe:** hospital; calendar year; delivery day of week; seasonality;
- **Maternal health factors**: maternal age; hypertension/diabetes; chorionic villious sampling; number of obstetric checks in pregnancy; number of US scans in pregnancy; neonatal status;
- **Child's fragility factors:** multiple birth; ICU admission
- **Child's size factors:** gestational age; birthweight; placenta weight;
- **Obstetric history factors:** number of previous liverbirths; number of previous CS;
- **Socio-demographic factors**: father's age; mother's nationality; mother's education; mother's occupation;
- **Obstetric factors**: eclampsia/pre-eclampsia; polyhydramnios; non-reassuring fetal status; placenta previa/abruptio placenta/ ante-partum haemorrhage; PROM; labour mode; labour analgesia; Rh iso-immunization; congenital malformaitons at birth

IVD:

- **Health care setting and timeframe:** hospital; calendar year; delivery day of week;
- **Maternal health factors:** mother's age; neonatal status; any medical assisted fertilization;
- **Child's fragility factors:** Apgar score at 5 minutes; multiple birth;
- **Child's size factors:** gestational age; placenta weight;
- **Obstetric history factors:** number of previus livebirths;
- **Socio-demographic factors:** father's age; mother's educational level;
- **Obstetric factors**: eclampsia/pre-eclampsia; PROM; labour mode; labour analgesia; presentation; Rh iso-immunization; placental secondment.

Supplementary Table 1b. Multivariable logistic regression model for the probability of length of stay (LoS) > ED for spontaneous vaginal deliveries (SVD, 2 days cutoff) and instrumental vaginal deliveries (IVD, 3 days cutoff). Hospital estimates (Odds ratio, OR) with 95% confidence interval (95%CI); Benjamini Hochberg (BH) p-value set at 5% discovery rate (bottom of each cell). Obs.= complete (case analysis) observations.

**HOSPITAL**	**DELIVERY MODE**<br>**OR (95%CI); with BH p value**	
	**SVD** (72,786 obs.)<br>**LoS > 2 vs. LoS ≤ 2**	**IVD** (7,020 obs.)<br>**LoS > 3 vs. LoS ≤ 3**
**A**	Reference	Reference
**B**	91.48 (80.23; 104.32)<br>0	8.66 (7.05; 10.63)<br>1.16E-92
**C**	5.02 (4.66; 5.41)<br>0	NS
**D**	27.94 (23.56; 33.14)<br>0	9.03 (5.85; 13.93)<br>7.43E-22
**E**	8.35 (7.62; 9.14)<br>0	2.36 (1.78; 3.13)<br>1.89E-08
**F**	2.89 (2.65; 3.16)<br>6.67E-127	NS
**G**	0.80 (0.75; 0.86)<br>1.44E-09	NS
**H**	2.84 (2.66; 3.02)<br>1.78E-223	1.60 (1.27; 2.03)<br>0.0004
**I**	10.52 (9.57; 11.55)<br>0	2.98 (2.26; 3.92)<br>1.10E-13
**J**	2.38 (2.23; 2.54)<br>7.27E-146	2.81 (2.24; 3.52)<br>4.40E-18
**K**	10.40 (9.55; 11.34)<br>0	2.53 (1.99; 3.52)<br>2.75E-13

Results of Supplementary Tables 1a and 1b belong to the same model of multivariable logistic regression models, adjusted for the following factors:

SVD:

- **Health care setting and timeframe:** hospital; calendar year; delivery day of week; seasonality
- **Maternal health factors**: maternal age; hypertension/diabetes; chorionic villous sampling; number of obstetric checks in pregnancy; number of US scans in pregnancy; neonatal status;
- **Child's fragility factors:** multiple birth; ICU admission
- **Child's size factors:** gestational age; birthweight; placenta weight;
- **Obstetric history factors:** number of previous liverbirths; number of previous CS;
- **Socio-demographic factors**: father's age; mother's nationality; mother's education; mother's occupation;
- **Obstetric factors**: eclampsia/pre-eclampsia; polyhydramnios; placenta previa/abruptio placenta/ ante-partum haemorrhage; non-reassuring fetal status; PROM; labour mode; obstructed labour; labour analgesia; Rh iso-immunization;

IVD:

- **Health care setting and timeframe:** hospital; calendar year; delivery day of week;
- **Maternal health factors:** mother's age; neonatal status; any medical assisted fertilization;
- **Child's fragility factors:** Apgar score at 5 minutes; multiple birth;
- **Child's size factors:** gestational age; placenta weight;
- **Obstetric history factors:** number of previus livebirths;
- **Socio-demographic factors:** father's age; mother's educational level;
- **Obstetric factors**: eclampsia/pre-eclampsia; PROM; labour mode; labour analgesia; presentation; Rh iso-immunization; placental secondment.

Supplementary Table 2a. Multivariable linear regression models for length of stay (linear endpoint) after spontaneous vaginal deliveries (SVD) and instrumental vaginal deliveries (IVD). Regression coefficients (RC) with 95% confidence interval (95%CI); Benjiamini Hochberg (BH) p-value set at 5% discovery rate (bottom of each cell). obs.= complete (case analysis) observations.

FACTORS (Reference category)	CLASSES	RC (95%CI) BH p-value	
		SVD (72,910 obs.)	IVD (7,154 obs.)
Calendar year (2005-2015)	Linear term	-0.01 (-0.01; -0.01) 6.74E-16	-0.02 (-0.03; -0.01) 0.0001
Seasonality (Reference=June-August)	Sep-Nov	0.03 (0.01; 0.05) 0.0057	NS
	Dec-Feb	0.06 (0.04; 0.08) 2.18E-09	NS
	Mar-May	0.06 (0.04; 0.08) 2.82E-09	NS
Number of previous livebirths (Reference=0)	1	-0.43 (-0.45; -0.42) 0	-0.49 (-0.58; -0.40) 5.41E-25
	2	-0.57 (-0.60; -0.54) 0	-0.63 (-0.82; -0.44) 6.98E-10
	3	-0.64 (-0.70; -0.59) 3.17E-127	-0.76 (-1.18; -0.34) 0.0014
	4	-0.66 (-0.73; -0.58) 1.09E-64	-1.56 (-2.70; -0.42) 0.0189
Number of previous CS (Reference = 0)	1	0.15 (0.10; 0.19) 3.14E-10	NS
Mother nationality (Reference = Italian)	Non-EU	0.12 (0.10; 0.14) 3.16E-29	NS
Mother's education (Reference = University/more)	Primary/none	0.12 (0.06; 0.17) 6.91E-05	NS
	Jr. Secondary	0.05 (0.03; 0.07) 7.50E-06	NS
	Secondary	0.03 (0.01; 0.04) 0.0027	NS
Father's age (Reference = 30-34 years)	20-24	-0.06 (-0.11; -0.02) 0.0106	NS
	25-29	-0.03 (-0.05; -0.01) 0.0185	NS
Mother's age (Reference = 20-24 years)	25-29	0.04 (0.01; 0.07) 0.0091	NS
	30-34	0.09 (0.06; 0.12) 5.96E-09	NS
	35-39	0.17 (0.13; 0.20) 2.17E-22	NS
	40-44	0.24 (0.20; 0.29) 4.03E-28	0.26 (0.12; 0.41) 0.0016
	45+	0.39 (0.23; 0.56) 5.41E-06	NS
Gestational age (Reference = 37-40 weeks)	29-32	-0.16 (-0.31; -0.01) 0.0454	NS
	33-36	0.50 (0.47; 0.54) 5.39E-135	0.59 (0.40; 0.78) 4.43E-09
	41+	-0.04 (-0.06; -0.03) 4.06E-06	NS

Birthweight<br><br>(Reference = 2.5-4.0 Kg)	< 2.0	-0.21 (-0.33; -0.09)<br>0.0013	NS
	2.0-2.5	0.36 (0.32; 0.41)<br>1.99E-52	0.27 (0.07; 0.47)<br>0.0206
	≥ 4.0	0.08 (0.06; 0.11)<br>1.01E-08	NS
Multiple birth<br>(Reference = singleton)	Twins or more	0.52 (0.38; 0.65)<br>2.13E-13	0.80 (0.27; 1.32)<br>0.0098
Placenta weight<br>(Reference = 500-999g	< 500	0.03 (0.01; 0.04)<br>0.0110	NS
	600-999	0.02 (0.01; 0.04)<br>0.0059	NS
	1,000-1,500	0.15 (0.03; 0.27)<br>0.0185	0.63 (0.16; 1.11)<br>0.0214
Hypertension/diabetes<br>(Reference = No)	Yes	0.19 (0.13; 0.25)<br>1.82E-09	NS
Pre delivery LoS (days)<br>(Reference <3)	6+	0.14 (0.05; 0.23)<br>0.0027	NS
Eclampsia/pre-eclampsia<br>(Reference = No)	Yes	1.03 (0.93; 1.14)<br>1.95E-86	0.90 (0.61; 1.19)<br>6.84E-09
Number of US scans in pregnancy (Reference <4)	6+	0.03 (0.01; 0.05)<br>0.0142	NS
RH iso-immunization<br>(Reference = No)	Yes	0.74 (0.48; 1.00)<br>5.60E-08	NS
Placenta previa*<br>(Referece=No)	Yes	0.27 (0.11; 0.44)<br>0.0020	
Labour mode<br>(Reference=Spontaneous)	Induced	0.15 (0.13; 0.17)<br>7.29E-44	0.11 (0.04; 0.18)<br>0.0067
	Augmented	0.10 (0.07; 0.13)<br>3.26E-11	NS
Obstructed labour<br>(Reference=No)	Yes	0.30 (0.18; 0.42)<br>1.32E-06	NS
Labour analgesia<br>(Reference = No)	Yes	NS	0.09 (0.03; 0.15)<br>0.0136
ICU admission<br>(Reference = No)	Yes	-0.14 (-0.20; -0.09)<br>7.02E-07	NS
Neonatal status<br>(Reference = Liveborn)	Stillborn	-1.68 (-1.83; -1.52)<br>3.80E-94	-0.82 (-1.47; -0.16)<br>0.0285
PROM<br>(Reference = No)	Yes	0.02 (0.00; 0.05)<br>0.0336	0.11 (0.03; 0.20)<br>0.0214
Non reassuring fetal status<br>(Reference = No)	Yes	0.57 (0.44; 0.70)<br>8.03E-17	NS
Apgar score at 5 minutes<br>(Reference = 8+)	< 8	0.12 (0.06; 0.20)<br>0.0017	0.21 (0.06; 0.35)<br>0.0136
Cord prolapse<br>(Reference=No)	Yes	1.64 (0.60; 2.68)<br>0.0033	NS
Placental Secondment<br>(Reference=Spontaneous)	Manual/<br>Instrumental	0.65 (0.59; 0.71)<br>1.78E-102	0.28 (0.15; 0.41)<br>0.0002
Congenital Malformations at Birth: Present	Absent	0.12 (0.06; 0.18)<br>0.0003	

* placenta previa/abruptio placenta/ ante-partum haemorrhage

Results of Supplementary Tables 2a and 2b belong to the same model of multivariable logistic regression models, adjusted for the following factors:

SVD:

- **Health care setting and timeframe:** hospital; calendar year; delivery day of week; seasonality
- **Maternal health factors**: maternal age; hypertension/diabetes; number US scans during pregnancy; neonatal status; pre-delivery LoS;
- **Child's fragility factors:** multiple birth; Apgar score at 5 minutes; ICU admission
- **Child's size factors:** gestational age; birthweight; placenta weight;
- **Obstetric history factors:** number of previous livebrths; number of previous CS;
- **Socio-demographic factors**: paternal age; mother's nationality; mother's education;
- **Obstetric factors**: eclampsia/pre-eclampsia; non reassuring fetal status; PROM; Rh iso-immunization; placenta previa/abruptio placenta/ ante-partum haemorrhage; labour mode; obstructed labour; labour analgesia; cord prolapse; plancental secondment; congenital malformations at birth.

IVD:

- **Health care setting and timeframe:** hospital; calendar year; number of admissions on delivery day
- **Maternal health factors**: maternal age; hypertension/diabetes; neonatal status; any medical assisted fertilization;
- **Child's fragility factors:** Apgar score at 5 minutes; multiple birth;
- **Child's size factors:** gestational age; birthweight; placenta weight;
- **Obstetric history factors:** number of previous livebrths; number of previous CS;
- **Socio-demographic factors**: mother's nationality; mother's educational level;
- **Obstetric factors**: eclampsia/pre-eclampsia; polyhydramnios; presentation; non-reassuring fetal status; placenta previa/ abruptio placenta/ ante-partum haemorrhage; PROM; presentation; labour mode; labour analgesia; placental secondment

Supplementary table 2b. Multivariable linear regression models for length of stay (linear endpoint) after spontaneous vaginal deliveries (SVD) and instrumental vaginal deliveries (IVD). Hospital estimates (Regression coefficients, RC) with 95% confidence interval (95%CI); Benjamini Hochberg (BH) p-value set at 5% discovery rate (bottom of each cell). obs.= complete (case analysis) observations

HOSPITAL	DELIVERY MODE RC (95%CI); with BH p value	
	**SVD** (72,910 obs.)	**IVD** (7,154 obs.)
**A**	Reference	Reference
**B**	1.10 (1.07; 1.12)<br>0	0.83 (0.73; 0.92)<br>4.10E-61
**C**	0.39 (0.36; 0.41)<br>2.53E-158	NS
**D**	0.95 (0.91; 0.99)<br>0	0.88 (0.66; 1.11)<br>2.62E-13
**E**	0.64 (0.61; 0.67)<br>0	0.68 (0.55; 0.82)<br>1.29E-21
**F**	0.26 (0.23; 0.30)<br>1.10E-48	NS
**G**	NS	-0.22 (-0.33; -0.11)<br>0.0002
**H**	0.31 (0.29; 0.34)<br>1.38E-128	0.15 (0.05; 0.26)<br>0.0136
**I**	0.67 (0.64; 0.70)<br>0	0.55 (0.41; 0.69)<br>1.14E-13
**J**	0.44 (0.42; 0.47)<br>5.70E-232	0.39 (0.28; 0.50)<br>3.18E-11
**K**	0.65 (0.62; 0.68)<br>0	0.43 (0.32; 0.55)<br>2.38E-12

Results of Supplementary Tables 2a and 2b belong to the same model of multivariable logistic regression models, adjusted for the following factors:

SVD:

- **Health care setting and timeframe:** hospital; calendar year; delivery day of week; seasonality
- **Maternal health factors**: maternal age; hypertension/diabetes; number US scans during pregnancy; neonatal status; pre-delivery LoS;
- **Child's fragility factors:** multiple birth; Apgar score at 5 minutes; ICU admission
- **Child's size factors:** gestational age; birthweight; placenta weight;
- **Obstetric history factors:** number of previous livebrths; number of previous CS;
- **Socio-demographic factors**: paternal age; mother's nationality; mother's education;
- **Obstetric factors**: eclampsia/pre-eclampsia; oligohydramnios; polyhydramnios; non reassuring fetal status; PROM; Rh iso-immunization; labour mode; obstructed labour; placenta previa/abruptio placenta/ ante-partum haemorrhage; labour analgesia; cord prolapse; plancental secondment; congenital maformations at births.

IVD:

- **Health care setting and timeframe:** hospital; calendar year; number of admissions on delivery day; seasonality;
- **Maternal health factors**: maternal age; hypertension/diabetes; neonatal status; any medical assisted fertilization;
- **Child's fragility factors:** Apgar score at 5 minutes; multiple birth;
- **Child's size factors:** gestational age; birthweight; placenta weight;
- **Obstetric history factors:** number of previous livebrths; number of previous CS;
- **Socio-demographic factors**: mother's nationality; mother's educational level;
- **Obstetric factors**: eclampsia/pre-eclampsia; polyhydramnios; presentation; non-reassuring fetal status; placenta previa/ abruptio placenta/ ante-partum haemorrhage; PROM; presentation; labour mode; labour analgesia; placental secondment

Supplementary Table 3. Adjusted[&] mean of length of hospital stay (LoS, in days) ± standard deviation (SD), by vaginal delivery mode (SVD vs. IVD), level of pregnancy risk (high vs. low), nationality of the mother (EU vs. non-EU) and maternity centre. EU= European Union; SVD= Spontaneous Vaginal Delivery; IVD=Instrumental Vaginal Delivery.

FACTORS	CLASSES	LoS ± SD (days)			
		SVD		IVD	
		Low risk pregnancies*	High Risk pregnancies#	Low risk pregnancies*	High Risk pregnancies#
Mother's nationality	EU	2.7 ± 0.4	3.0 ± 0.5	3.1 ± 0.4	3.4 ± 0.5
	Non-EU	2.8 ± 0.4	3.1 ± 0.5	3.3 ± 0.4	3.4 ± 0.5
Hospital	A	2.3 ± 0.2	2.5 ± 0.3	2.8 ± 0.3	3.1 ± 0.3
	B	3.4 ± 0.2	3.6 ± 0.3	3.6 ± 0.3	3.9 ± 0.3
	C	2.7 ± 0.2	2.8 ± 0.3	3.0 ± 0.2	3.2 ± 0.3
	D	3.3 ± 0.2	3.4 ± 0.3	3.5 ± 0.2	3.8 ± 0.3
	E	3.0 ± 0.2	3.1 ± 0.3	3.6 ± 0.2	3.7 ± 0.3
	F	2.6 ± 0.2	2.7 ± 0.3	2.8 ± 0.2	3.0 ± 0.3
	G	2.3 ± 0.2	2.5 ± 0.3	2.6 ± 0.2	2.8 ± 0.3
	H	2.6 ± 0.2	2.8 ± 0.3	3.0 ± 0.2	3.2 ± 0.3
	I	3.0 ± 0.2	3.1 ± 0.3	3.4 ± 0.2	3.5 ± 0.5
	J	2.8 ± 0.2	2.9 ± 0.3	3.1 ± 0.3	3.4 ± 0.4
	K	2.9 ± 0.2	3.1 ± 0.3	3.2 ± 0.3	3.5 ± 0.3

[&] Mean LoS adjusted for all factors displayed at the bottom of Supplementary Tables 2a and 2b, for both SVD and IVD.

- **pregnancies simultaneously meeting all the following conditions:**
  No hypertension/diabetes of the mother; No assisted medical fertilization; singleton pregnancies; No eclampsia/pre-eclampsia; cephalic presentation; livebirth; pre-delivery LoS < 3 days; No oligohydramnios; No polyhydramnios; Apgar score at 5 minute ≥ 8; Gestation 37-40 weeks; Birthweight 2.500-4.000 g; Placenta weighing 500-599g; Spontaneous labour; No administration of labour analgesia; No fetal distress; No obstructed labour; No PROM; Spontaneous placental secondment; No cord prolapse; No Rh iso-immunization; no congenital malformations at birth.

pregnancies meeting <u>AT LEAST ONE</u> of the following conditions:
Any assisted medical fertilization; Multiple births; Eclampsia/pre-eclampsia; Breech/Shoulder presentation; Stillbirth; Pre-delivery LoS ≥ 3 days; Oligohydramnios; Polyhydramnios; Apgar score at 5 minute < 8; Gestation <37 or ≥ 41 weeks; Birthweight <2,5 or ≥ 4.0 Kg; Placenta weighing <500g or ≥600g; Labour induced or augmented; Administration of labour analgesia; Non-reassuring fetal status; Obstructed labour; PROM; Manual/instrumental placental secondment; Cord prolapse; Rh iso-immunization; congenital malformations at birth.
